# Peat-based hairy root transformation using *Rhizobium rhizogenes* as a rapid and efficient tool for easily exploring potential genes related to root-knot nematode parasitism and host response

**DOI:** 10.1186/s13007-023-01003-3

**Published:** 2023-03-04

**Authors:** Xu Zhang, Shihui Li, Xin Li, Mengyuan Song, Si Ma, Yongqiang Tian, Lihong Gao

**Affiliations:** Beijing Key Laboratory of Growth and Developmental Regulation for Protected Vegetable Crops, College of Horticulture, China Agricultural University, 2 Yuanmingyuan Xilu, Yuanmingyuan West Road No.2, Haidian District, Beijing, 100193 People’s Republic of China

**Keywords:** *Rhizobium rhizogenes*, Hairy root, Cucumber, Plant-parasitic nematode, Transgenic root

## Abstract

**Background:**

Root-knot nematodes (RKNs) pose a worldwide threat to agriculture of many crops including cucumber. Genetic transformation (GT) has emerged as a powerful tool for exploration of plant-RKN interactions and genetic improvement of RKN resistance. However, it is usually difficult to achieve a highly efficient and stable GT protocol for most crops due to the complexity of this process.

**Results:**

Here we firstly applied the hairy root transformation system in exploring root-RKN interactions in cucumber plants and developed a rapid and efficient tool transformation using *Rhizobium rhizogenes* strain K599. A solid-medium-based hypocotyl-cutting infection (SHI) method, a rockwool-based hypocotyl-cutting infection (RHI) method, and a peat-based cotyledon-node injection (PCI) method was evaluated for their ability to induce transgenic roots in cucumber plants. The PCI method generally outperformed the SHI and RHI methods for stimulating more transgenic roots and evaluating the phenotype of roots during nematode parasitism. Using the PCI method, we generated the CRISPR/Cas9-mediated malate synthase (*MS*) gene (involved in biotic stress responses) knockout plant and the *LATERAL ORGAN BOUNDARIES-DOMAIN 16* (*LBD16*, a potential host susceptibility gene for RKN) promoter-driven *GUS* expressing plant. Knockout of *MS* in hairy roots resulted in effective resistance against RKNs, while nematode infection induced a strong expression of *LBD16*-driven *GUS* in root galls. This is the first report of a direct link between these genes and RKN performance in cucumber.

**Conclusion:**

Taken together, the present study demonstrates that the PCI method allows fast, easy and efficient in vivo studies of potential genes related to root-knot nematode parasitism and host response.

**Supplementary Information:**

The online version contains supplementary material available at 10.1186/s13007-023-01003-3.

## Background

Plant-parasitic nematodes (PPNs) are globally ubiquitous pathogens that cause significant damage to a wide variety of crops [1]. Among various PPNs, root-knot nematodes (RKNs) (*Meloidogyne* spp.) are usually the most damaging to agriculture because they have a broad host range and parasitize almost every species of vascular plant [2, 3]. The RKNs are obligatory sedentary endoparasites that infect host plant roots by their second-stage infective juveniles (J2) through establishing a permanent feeding site, which consists of several multinucleate giant (MG) cells [4]. These MG cells function as specialized sinks supplying the sole sources of nutrients for the growth and development of RKNs throughout their reproductive life cycle. To date, RKNs have been reported to reproduce on thousands of cultivated and wild plants, causing approximately tens of billions of dollars in economic losses every year [5]. Since most crop plants are sensitive to RKN infection, it is extremely important to develop potential approaches to enhance plant resistance towards RKNs.

Reaction of a plant to RKN parasitism is most closely associated with the plant species and cultivar, because plant responses and tolerance to biotic stresses are driven to a large extent by genetic differentiation among plants [6]. Theoretically, for a given plant species or cultivar, it is possible to enhance its resistance to RKN by modifying the plant through genetic engineering [7, 8]. In the past decade, *Agrobacterium tumefaciens*-mediated genetic transformation (GT) has emerged as a powerful tool for genetic improvement of PPN resistance in crop plants [9–11]. *A. tumefaciens* is a soil microorganism that is capable of infecting a broad assortment of plants at wound sites and thereafter producing crown gall tumors by transferring a specific segment (T-DNA) of the tumor-inducing (Ti) plasmid into the plant nuclear genome [12]. Since the bacterial genes on the T-DNA are not necessary for its transfer, the Ti plasmid can be modified by deleting these genes and replacing them with genes of interest [13]. Based on this mechanism, it is theoretically possible to transfer any gene related to RKN resistance into the chromosomes of the plant cell. So far, GT systems for several model plants and crops (e.g. tomato, Arabidopsis, soybean and rice) have been successfully applied to examine the plant-RKN interaction and thereby facilitate plant genetic modifications towards RKN-resistance [14–17]. Despite the success, it is usually difficult to achieve a highly efficient and stable GT protocol for most crops due to the labor-intensive and time-consuming features as well as the complexity of this process (e.g. plant recalcitrance to regeneration, and poor transformability), strongly hampering research on the molecular basis of the plant-RKN interaction [18].

*Rhizobium rhizogenes* (previously known as *Agrobacterium rhizogenes*), a relative of* A. tumefaciens*, can induce hairy roots upon wounding and infection of the stem or leaf tissues in monocot and eudicot plants [19]. Its root-inducing (Ri) plasmid, which can be modified with a target gene, is responsible for the stable introduction of genetic material into the nucleus of the host plant cell. Hairy roots induced by *R. rhizogenes* are anatomically and metabolically similar to normal roots, and constitute a valuable tool in plant functional biology, metabolic engineering, molecular pharming, biotechnology, and analyses of rhizosphere physiology and biochemistry [20–22]. Since *R. rhizogenes*-mediated hairy root transformation avoids restrictive and complex tissue culture steps [23], it has been successfully used to generate transgenic composite plants against fastidious pathogens (e.g. *Fusarium oxysporum* spp., *Candidatus* Liberibacter spp., and common cutworm) [20, 24, 25]. In addition, it has recently been applied to investigate the interaction of RKNs with several crop plants including grape, soybean and peanut [26–28]. Despite this, most crops still lack a rapid and efficient hairy root transformation system for easily exploring interactions between plant root and RKNs.

Cucumber (*Cucumis sativus* L.) is one of the most economically important vegetable crops worldwide and makes a substantial nutritional contribution to the human diet [29]. Root knot induced by the RKN *Meloidogyne incognita* is the most economically important cucumber disease around the world. Unfortunately, however, the conventional hybridization breeding method cannot confer *M. incognita* resistance to cucumber, because almost all cucumber cultivars are susceptible to *M. incognita* [30, 31] and lack candidate resistance genes [29]. Although *A. tumefaciens*-mediated GT system has been developed for cucumber, it is time-consuming and labor-intensive and shows extremely low transformation ratio [32, 33]. In light of this, we developed a rapid and efficient tool for easily exploring root-RKN interactions in cucumber plants, based on *R. rhizogenes*-mediated hairy root transformation using a peat-based cotyledon-node injection (PCI) method. Using the PCI method, we provided the first direct experimental evidence for the critical roles played by the malate synthase (*MS*) gene, and analyzed the response of the *LOB-domain protein 16* (*LBD16*, a potential host susceptibility gene for RKN) during RKN parasitism in cucumber. Our study demonstrates that the PCI method allows fast, easy and efficient in vivo studies of exploring potential genes related to root-knot nematode parasitism and host response.

## Materials and methods

### Plant material, nematodes and *R. rhizogenes* strain

Cucumber (*Cucumis sativus* L.) cultivar “Xintaimici” was used in this study. Seeds were surface sterilized using 4% sodium hypochlorite and germinated on moistened filter paper in darkness for the RHI and the PCI methods. For the SHI method, seeds were surface sterilized and germinated on MS solid medium. *M. incognita* race 2 was maintained on cucumber in sterilized soil. The egg masses were collected and sterilized with 0.5% sodium hypochlorite for 3 min and then submerged in sterile water at 25 ℃ for 3 days. Freshly hatched pre-J2s were collected using a 500-mesh screen and stored in 4 ℃ before infection.

*R. rhizogenes* strain K599 (Weidi Biotechnology, China) was used in this study to induce hairy roots which harboring plasmid pRi2659 (agropine type) and had a wide range of hosts including Cucurbitaceae.

### Construction of CRISPR/Cas9 vector and *pCsLBD16::GUS* vector

*CRISPR/Cas9* genome editing construct for *CsMS* editing was generated using the pHEE401E vector tagged with *GFP*. Two sgRNAs were driven by the U6 promoter, and the *Cas9* protein was driven by the egg cell-specific promoter [34]. The empty pHEE401E vector was used as a control. Two sgRNAs were targeted against cucumber malate synthase gene (CsaV3_1G009520) designed using CRISPR-GE tool (http://skl.scau.edu.cn/) [35]. For analysis the activity of *CsLBD16*(*LATERAL ORGAN BOUNDARIESDOMAIN*, Csa3G398920) which homolog of the Arabidopsis *LATERAL ORGAN BOUNDARIES-DOMAIN 16* (*AtLBD16,* At2g42430) under nematode parasitism, the vector pCAMBIA1391 carrying *GUS* gene was driven by the 2000 bp promoter region of the *CsLBD16* gene, generating a *pCsLBD16::GUS* recombinant construct. The primer's sequences for construction of two vectors are listed in Table 1.

**Table 1:** List of primers used in this study

Primer name	Application	Primer (5'–3')
q*CsMS*-F	Relative expression	GCCTTGTTGTTTGTCGCTGA
q*CsMS*-R		TTAGTCGCCGGATCAAACCC
q*CsLBD16*-F		CAGAAACCCTAATGGATTCAGGAAG
q*CsLBD16*-R		GTGGGCTTGGGTTGTTCGTAATTTG
q*CsTUB*-F		CATTCTCTCTTGGAACACACTGA
q*CsTUB*-R		TCAAACTGGCAGTTAAAGATGAAA
*pCsLBD16*-F	pCAMBIA-1391	GCGCGCCAAGCTTGGCTGCAGACCTAAGTCCGAAGCCATAAGTGAC
*pCsLBD16*-R		TCTTAGAATTCCCGGGGATCCGGGAAAATAGAAGAAATGGCCGTGC
*CsMS*DT1-BsF	*CsMS*-pHEE401E	ATATATGGTCTCGATTGAGAGGCTACGACGTTCCAGGTT
*CsMS*DT1-F0		TGAGAGGCTACGACGTTCCAGGTTTTAGAGCTAGAAATAGC
*CsMS*DT2-R0		AACTGCTAATTTTCGACGCTCTCAATCTCTTAGTCGACTCTAC
*CsMS*DT2-BsR		ATTATTGGTCTCGAAACTGCTAATTTTCGACGCTCTCAA
*GFP*-F	*GFP* characterization	CAAGGGCGAGGAGCTGTTCACCG
*GFP*-R		CAGCTCGTCCATGCCGTGAGTGA
*CsMSCsa9*-F	Mutant characterization	GCTTGGGATGTATTCCGAATCA
*CsMSCsa9*-R		GGATGAAGATTTACCTGGAGTG

### Three methods of transgenic hairy roots development system

Three methods were carried out to develop transgenic hairy roots. *R. rhizogenes* strain K599 harboring empty vector pCAMBIA 1305-*GFP* was used for transgenic hairy roots screening. Single colony was collected and cultured for 6 h in LB broth at 28 ℃ for the K599 collection in three methods.

- (i) For solid-medium-based hypocotyl-cutting infection (SHI) method, surface-sterilized seeds were germinated on solid MS medium. And then bacterial solution was plated onto solid LB medium with 50 mg/L streptomycin and 50 mg/L kanamycin. Seedlings with unfolded cotyledons were cut obliquely at the junction of the hypocotyl and the root system and dipped into the bacterial mass on solid medium. Transfer seedlings to the MS solid medium containing 200 mg/L cephalosporin on a sterilized filter and cover another sterilized filter paper on the wounding site to maintain humidity and prevent excessive growth of the bacterial solution. Transferred the infected seedlings every 3 days until the hairy roots developed well at the infection sites and checked the transgenic hairy roots under stereomicroscope.
- (ii) For the rockwool-based hypocotyl-cutting infection (RHI) method, surface-sterilized seeds were germinated on sterilized filter paper and sowed into a rockwool seedling block. A single colony was picked and placed in 20 ml LB liquid medium plus 50 mg/L streptomycin and 50 mg/L kanamycin at 28 ℃ (180rpm) until the OD_600_ value reached to 0.6. The bacterial solutions were centrifuged at 4000 g for 10 min and then re-suspended in 10 ml MS liquid medium. Cucumber seedlings with first expanded true leaf were cut off at the hypocotyl and transferred into a rockwool seedling block. The re-suspended K599 solution was inoculated into the wounding site and transformed in an artificial climate chamber. During the hairy root development, the rock-wool blocks were watered with nutrient solutions to maintain the growth of the seedlings.
- (iii) For peat-based cotyledon-node injection (PCI) method, surface-sterilized seeds were germinated on sterilized filter paper and sowed into a peat-based substrate block. At the same time, K599 solution were plated onto LB solid medium with antibiotic like SHI method. Germinated seeds were transferred into a peat-based seedling block and grew at artificial climate chamber until the cotyledons were exposed and turned green but still not expanded. The bacterial mass used in PCI method was collected from solid LB medium using a spreader by adding sterilized water. A micro syringe is used to inject the bacterial mass into cotyledon-node and create mechanical damage by the tip. Make sure the tip of syringe went through the central part of the pericycle. Then, the seedlings were transferred in the plug lid to maintain humidity and treated nutrient solution to keep normal growth of the seedlings.

In all three methods, the hairy roots started to emerge at about 14 days after inoculated with K599. The regeneration of the hairy root and the transgenic root induction efficiency was characterized by the proportion of hairy roots and transgenic hairy roots in the total inoculated plants [36].

### RNA, DNA extraction and Quantitative Real-Time PCR

Cucumber mock-infection and *M. incognita*-infection roots at different developmental stages were used for total RNA extraction and cDNA synthesis (Vazyme, China). RNA quantification was done using a Nanodrop 2000 (Thermo Fisher Scientific). The RT-qPCR analysis using SYBR Green Master Mix (Vazyme, China) was performed in ABI 6500 Real-Time PCR System (Applied Biosystems). *CsTUB* (accession number Csa4G000580) was used as internal control. Relative expression abundance of candidate genes was calculated with the formula 2^−ΔΔ^Ct. All reactions were performed with four biological replicates. Genomic DNA of hairy roots was extracted using the FastPure Plant DNA Isolation Mini Kit (Vazyme, China). All the primers for characterized *GFP* from hairy roots and RT-qPCR analysis are listed in the Table 1.

### Transgenic hairy roots characterization and target gene mutation detection

Hairy roots expressing *GFP* were easily visible under a fluorescent stereomicroscope (M165 FC; Leica). The injection site was hand-dissected at 7 days after inoculation and embedded in paraffin using a routine method. Genomic DNA was extracted from transgenic hairy roots to characterize the mutation of *CsMS* through gene specific primers. The purified PCR products was cloned using a pClone007 Versatile Simple Vector Kit (Tsingke, Beijing) and transformed in DH5α for sequencing. DNA fragments spanning both target sites were amplified by PCR using primers showed in Table 1.

### Nematode infection assays

In order to inoculate the transgenic hairy roots with *M.incognita* race 2, aqueous suspension of pre-J2s was standardized to about 300 J2s per ml sterilized water. Total length of transgenic hairy roots was scanned using a scanner (Epson, Japan) and analyzed using a software WinRHIZO (Regent Instruments, Inc., Canada). The number of nematodes inoculated in transgenic roots was recorded at different developmental stages using an acid-fuchsin staining method [37] and observed with an Olympus BX53 microscope (Olympus, Japan). The root galls and egg masses per mm roots in different transgenic hairy roots were counted to evaluate the nematode parasitism. To estimate the giant cells size in CRISPR/Cas9-guided *CsMS* knock-out hairy roots, representative galls were fixed and prepared by the paraffin-embedded method. The 6-μm sections were stained with 0.05% toluidine blue and photographed using an Olympus CX41 microscope. The image J software was used to measure the area of giant cells and total area of sections. Statistical analysis of the infection parameters and GC areas was performed using *t*-test in SPSS, significance was indicated by asterisks *(*P* < 0.05).

All the hairy roots were performed GUS staining and the transgenic hair roots showed high GUS signals were selected to analyze the expression of *pCsLBD16::GUS* at 7, 14, 21 and 28 DAI following the protocol of GUS staining kit (Obiolab, China). The mock-infection transgenic hair roots expressed the *GUS* staining paraffin-were embedded and sectioned into 6-μm slices for characterizing the *GUS* expression in lateral root primordia. Observation of histochemical *GUS* stained root galls were spread on a microscope slide and mounted in chloral hydrate solution. Moreover, fresh frozen sectioning was used to analysis tissue localization of *GUS* expression in root galls.

## Results

### Comparison of three methods for induction of transgenic hairy roots in cucumber

**Fig. 1:**
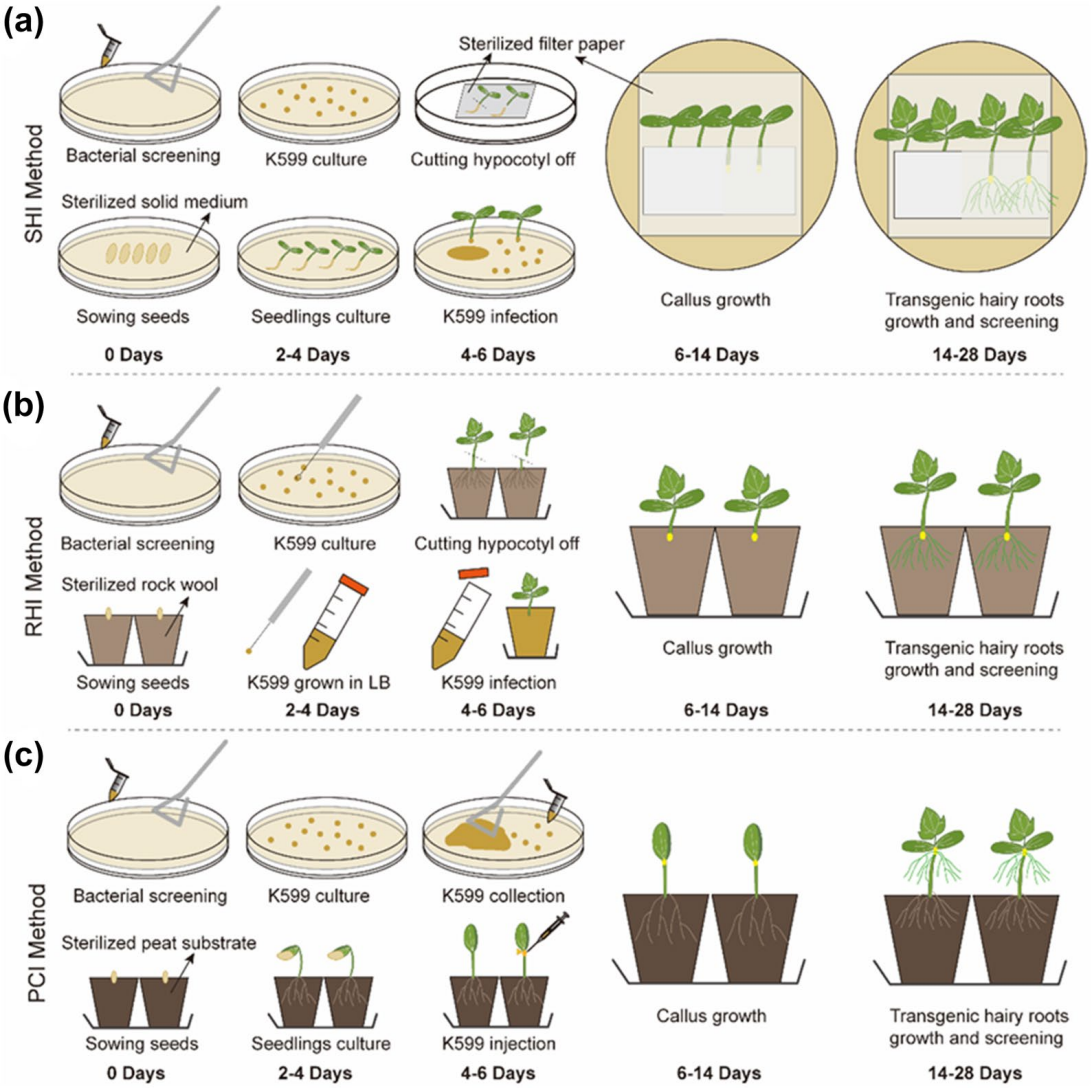
Three methods of *R. rhizogenes*-mediated transgenic hairy roots development for cucumber. **a** Procedure of solid-medium-based hypocotyl-cutting infection (SHI) method. Sterilized seeds were sowed on MS solid medium and then cut hypocotyl off for K599 infection. The sterilized filter paper was used to keep the humidity of the infected sites. **b** Procedure of rockwool-based hypocotyl-cutting infection (RHI) method. Sterilized seeds were sowed into sterilized rock wool and then cut the seedlings hypocotyl off for infection using the K599 solution. **c** Procedure of peat-based cotyledon-node injection (PCI) method. Sterilized seeds were sowed into sterilized peat substrate and the K599 mass collected from screening medium was injected into the cotyledon-node for inducing transgenic hairy roots

**Fig. 2:**
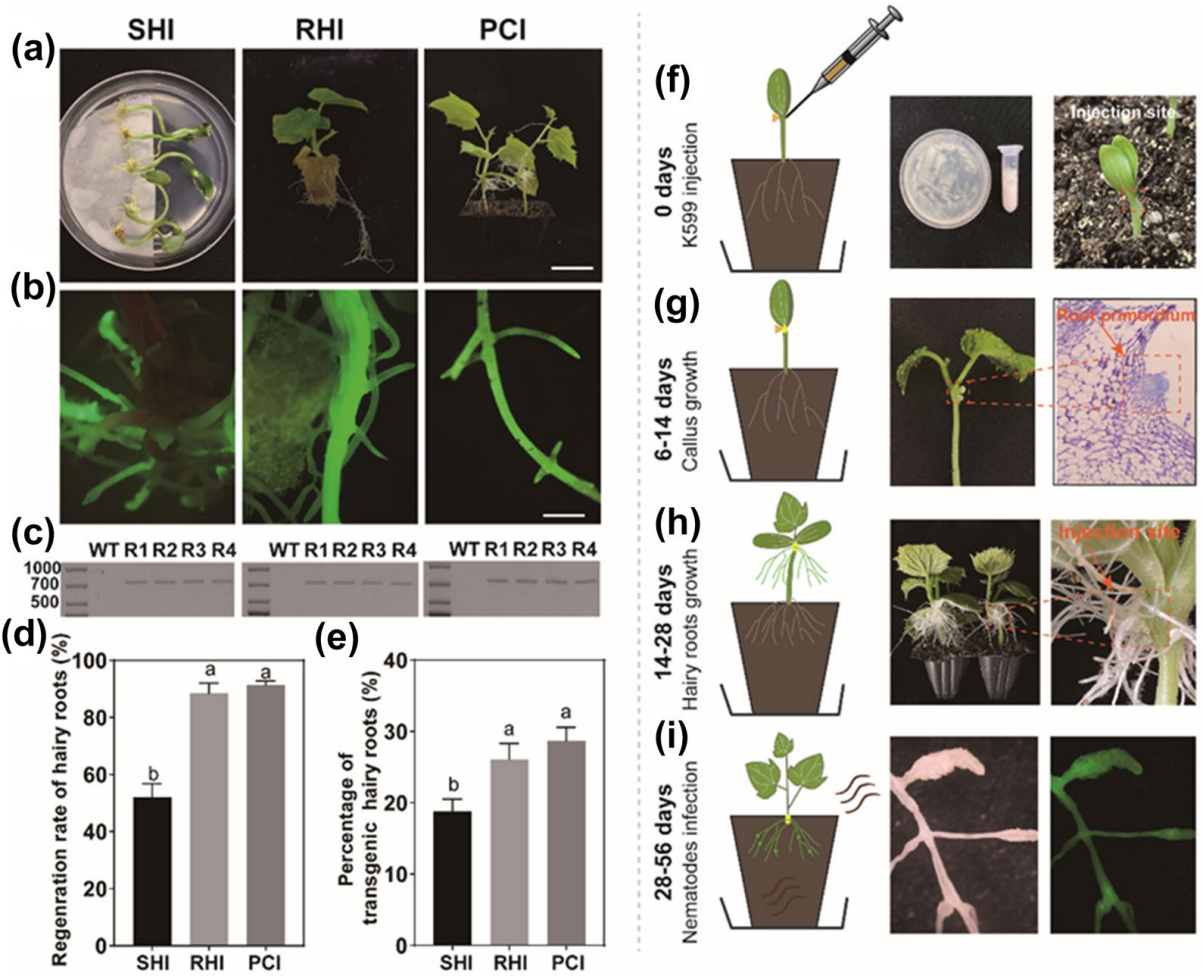
Selection of the optimal method to induce transgenic hairy roots and the process of hairy roots development using the PCI method. **a** Representative pictures of transgenic hairy roots development of three methods. Bar:5 cm **b** Transgenic hairy roots screening using *GFP* fluorescence under microscope. Bar:5 mm **c** RT-PCR analysis of the transgenic hairy roots for confirming the integration and expression of *GFP* reporter gene. **d** and **e** represent the regeneration rate for inducing hairy roots using three methods and the percentage of transgenic hairy roots induced using three methods respectively. The Histogram represent data from six independent experiments (25–60 replicates per condition in each method), The letters represent significant differences at *p* < 0.05 according to the LSD multiple comparisons test in different treatments. **f** K599 was collected from screening medium and injected into injected into the cotyledon-node. **g** Callus was induced at 6–14 day after injection and the root primordium was established. **h** The hairy roots were induced at the injection site and grew densely at about 14–28 days after injection. **i** Cut the original roots and infected with *M.incognita*

In this study, we compared three different methods (i.e. SHI, RHI and PCI) to induce transgenic hairy roots in cucumber using *R. rhizogenes* K599. For all three methods callus tissues appeared around the wounding site at approximately 6–14 days after inoculation with *R. rhizogenes* K599 and expanded gradually (Fig. 1a–c). Adventitious hairy roots generated from callus tissues at 14–28 days after inoculation and grew vigorously. As shown in Fig. 2, the hairy roots were induced in all three methods and showed green fluorescence under fluorescence microscope, indicating that the hairy roots have been transferred with the green fluorescent protein (GFP) tagged vector (Fig. 2a, b). Further test through RT-PCR confirmed that all three methods successfully introduced the GFP-labeled gene into the hairy roots (Fig. 2c). In spite of this, the RHI and PCI methods yielded higher (*P* < 0.05) regeneration rate of hairy roots than the SHI method and there was no significant (*P* > 0.05) difference between the RHI and PCI methods (Fig. 2d). A similar trend was observed for the percentage of transgenic roots (Fig. 2e). However, the RHI-induced hairy roots were tightly bound on the rockwool substrate (Fig. 2b), making it hard to sample the transgenic roots. In addition, the SHI method required a very strict sterile condition making this method inconvenient and labor-intensive. Thus, the PCI method overall outperformed the SHI and RHI methods for inducing transgenic hairy roots in cucumber.

### Procedure of the peat-based cotyledon-node injection (PCI) method

For the PCI method, bacterial mass collected from the screening medium was injected into unfolded cotyledon node using a 1-mL syringe (Fig. 2f). At 6–14 days after injection, the formation of calli appeared around the injection site (Fig. 2g). The tissue section at 8 days after injection showed that the root primordium had been established at the injection site (Fig. 2g). In order to ensure the survival and growth of adventitious hairy roots, seedlings injected with bacterial mass were covered with a plastic cap to keep high humidity. At 14–28 days after injection, the hairy roots generated from the injection site and developed intensively (Fig. 2h). After there were sufficient hairy roots, the original roots were cut off at 1 cm behind the injection site (Fig. 2h), and then the hairy roots were directly grown into the soil and treated with RKNs (Fig. 2i). The formation of root galls in transgenic hairy roots (Fig. 2i) indicated that the PCI method can satisfy the research on the interaction between roots and RKNs.

### Demonstration of *malate synthase *(*MS*) gene function using the PCI method

**Fig. 3:**
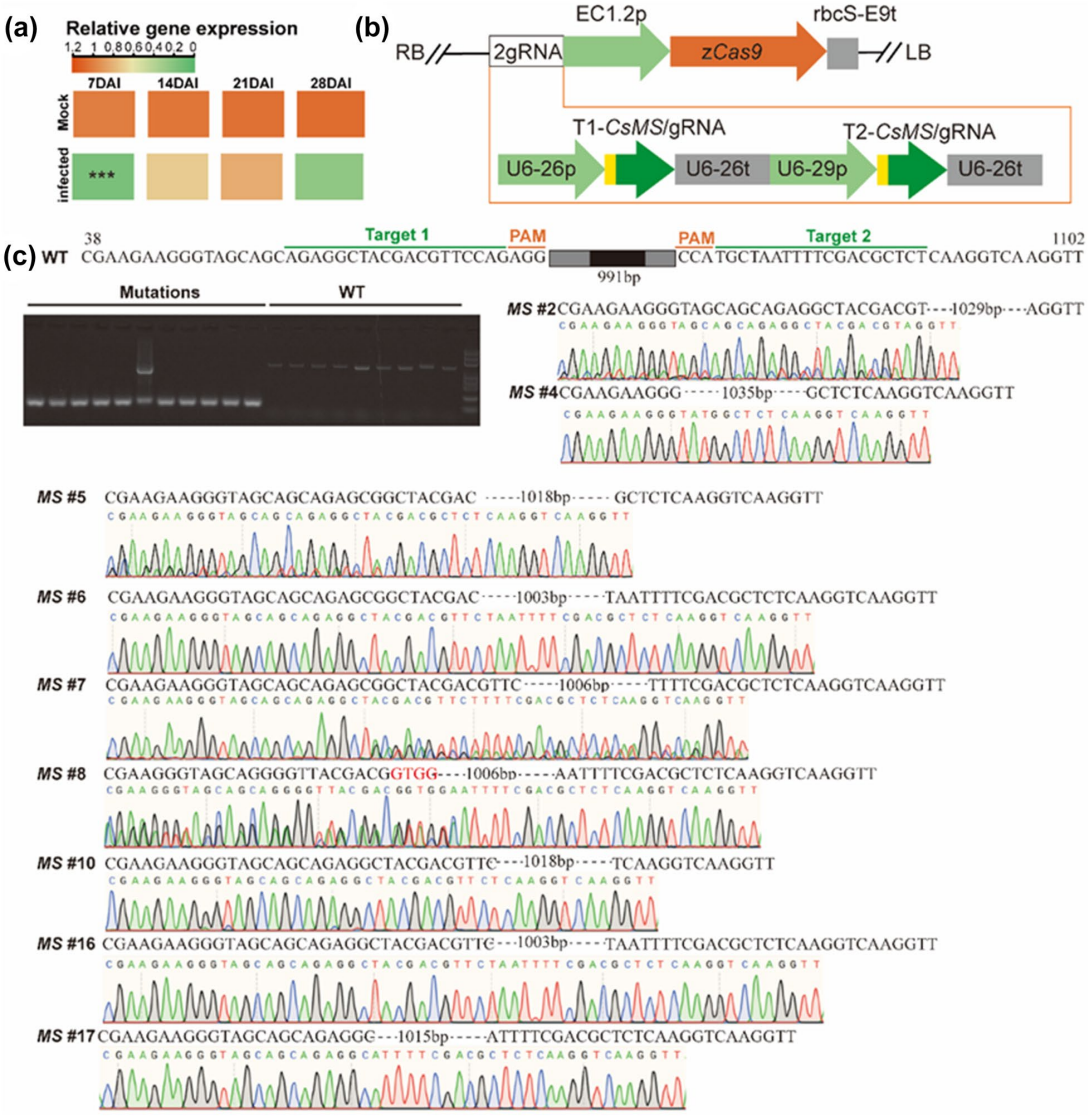
CRISPR/Cas9-guided gene editing in cucumber transgenic hairy roots using the PCI method. **a** Relative expression of *CsMS* gene in mock-infection and nematode-infection roots during nematode parasitism. Student’s *t*-test was used for statistical analysis. n = 4. ***P < 0.001. **b** Schematic diagram for constructing two sgRNA-expressing cassettes in the binary vector pHEE401E. **c** Mutation sequencing in the cucumber transgenic hairy roots after CRISPR/Cas9-mediated gene editing

Untargeted metabolomics of cucumber roots showed that RKN-infected roots had a significantly (*P* < 0.05) higher relative abundance of malic acid (MA) at 14 days after inoculation (DAI) with *M*. *incognita* J2 as compared with uninfected roots (data not shown). These response results were corroborated by decreased expression of the malate synthase (*MS*) gene in RKN-infected roots at 7 DAI (Fig. 3a), indicating that the glyoxylate cycle was probably required for RKN parasitism of roots. To assess the applicability of the PCI method to genome editing, we used CRISPR/Cas9-mediated mutagenesis to knock out *CsMS* in the hairy roots induced by the PCI method. In the vector, two sgRNAs were driven by the U6 promoter, and the Cas9 protein was regulated by the egg cell-specific promoter (EC1.2) (Fig. 3b). These two sgRNAs were designed against two DNA sites separated by 991 bp (Fig. 3c) using CRISPR-GE tool (http://skl.scau.edu.cn/). Sequencing results from both sides of the editing targets confirmed the successful editing of *CsMS* gene in the transgenic hairy roots of cucumber, the deletion of *CsMS* gene between two editing targets (Fig. 3c). These results demonstrate the feasibility of using the PCI method to generate targeted mutation in cucumber hairy roots.

**Fig. 4:**
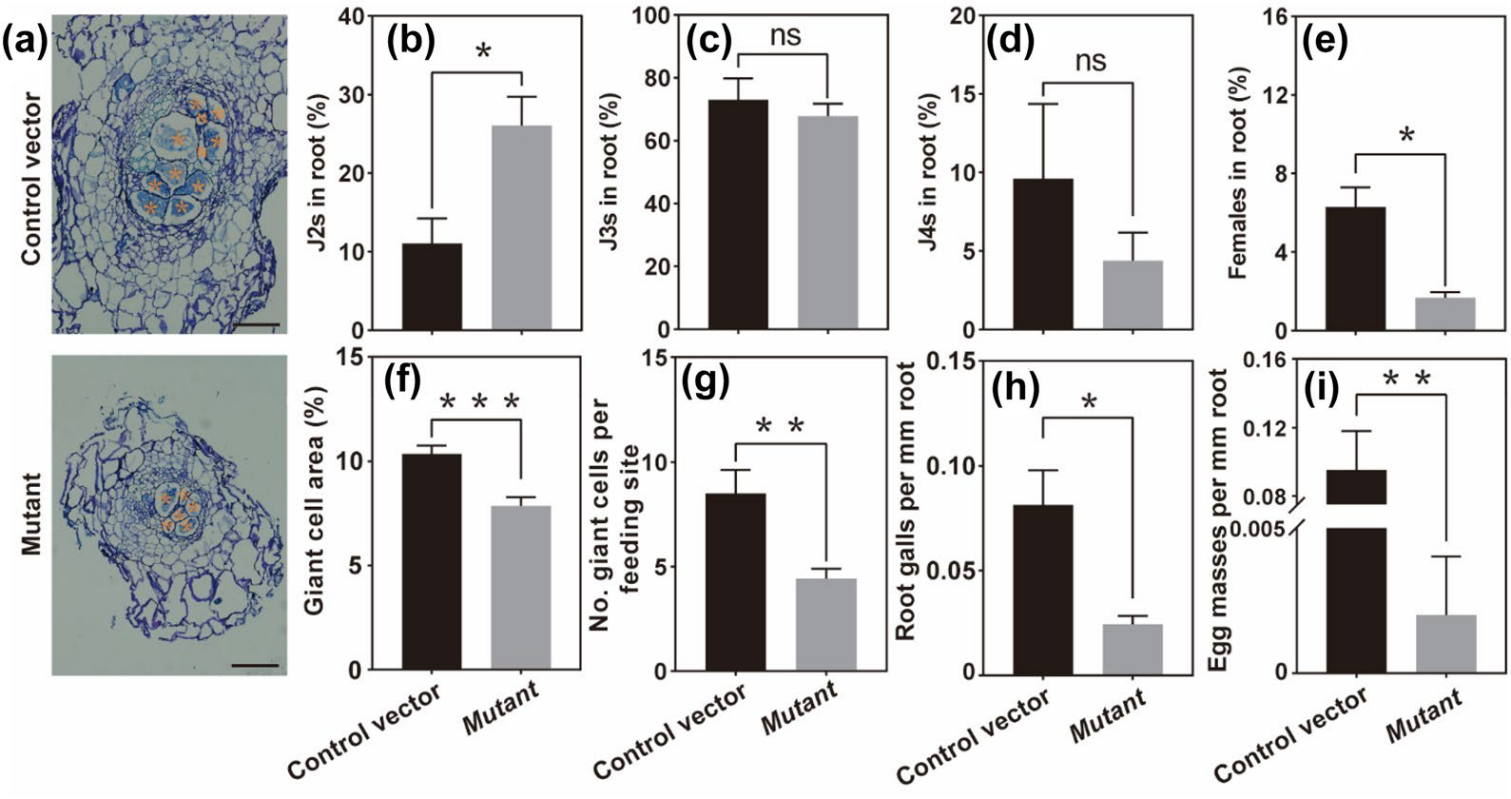
Editing of Malate synthase gene affected the establishment of feeding sites and the development of *M. incognita* in transgenic hairy roots. **a** Toluidine staining of paraffin Sects. (6-μm) of galls from the empty-vector control hairy roots *and CRISPR/Cas9-*guided *CsMS* knock-out hairy roots and photographed at 40 × magnification. Asterisks, GCs; N, Nematode; Bar:200 μm. **b**–**e** The proportion of J2(**b**), J3(**c**), J4(**d**) and female **e** nematodes to the total number of *M. incognita* in transgenic hairy roots at 14 DAI. **f** Proportion of giant cell area in root galls of transgenic hairy roots. **g** Numbers of giant cells per feeding site induced in transgenic hairy root. **h**–**i** Root galls and egg masses per mm of the empty-vector control hairy roots *and CRISPR/Cas9-*guided *CsMS* knock-out hairy roots after nematode infection. Data are presented as the mean ± SE, Student’s *t*-test, n ≥ 4. **p < 0.01; *p < 0.05

To further confirm the role of *CsMS* gene in RKN parasitism, we compared the ability of *M. incognita* to parasitize the empty-vector control and CRISPR/Cas9-guided *CsMS* knock-out hairy roots (Additional file 1: Figure S2). Histological observations of gall sections at 28 DAI showed that the *M. incognita* had initiated feeding sites composed of less giant cells in the CRISPR/Cas9-guided *CsMS* knock-out hairy roots as compared to the empty-vector control (Fig. 4a). In addition, the giant cells of the CRISPR/Cas9-guided *CsMS* knock-out hairy roots were generally smaller than those of the empty-vector control (Fig. 4a). The proportion of* M*. *incognita* J2 was significantly (*P* < 0.05) higher in the CRISPR/Cas9-guided *CsMS* knock-out hairy roots than in the empty-vector control (Fig. 4b). In contrast, the proportion of either *M*. *incognita* J3 or J4 was lower in the CRISPR/Cas9-guided *CsMS* knock-out hairy roots compared to the empty-vector control (Fig. 4c, d). Moreover, the CRISPR/Cas9-guided *CsMS* knock-out hairy roots had a significantly (*P* < 0.05) lower proportion of adult female *M*. *incognita* as compared to the empty-vector control (Fig. 4e). These results reveal that *M. incognita* exhibited a significant developmental delay in the CRISPR/Cas9-guided *CsMS* knock-out hairy roots. This was also corroborated by the histological observations of gall sections (Fig. 4a). Particularly, a significant (*P* < 0.001) reduction in the relative area of giant cells at the feeding sites of *M. incognita* was observed in the CRISPR/Cas9-guided *CsMS* knock-out hairy roots compared to the empty-vector control (Fig. 4f). A similar trend was observed in the number of giant cells per feeding site (Fig. 4g). Furthermore, the CRISPR/Cas9-guided *CsMS* knock-out hairy roots had a significantly lower relative abundance of either root galls (*P* < 0.05) or egg masses (*P* < 0.01) as compared to the empty-vector control (Fig. 4h, i). Taken together, these results show that the PCI method can be used efficiently for genome editing in plants and for the exploration of potential genes associated with RKN parasitism.

### Verification of the response of *LBD16* to nematode parasitism using the PCI method

**Fig. 5:**
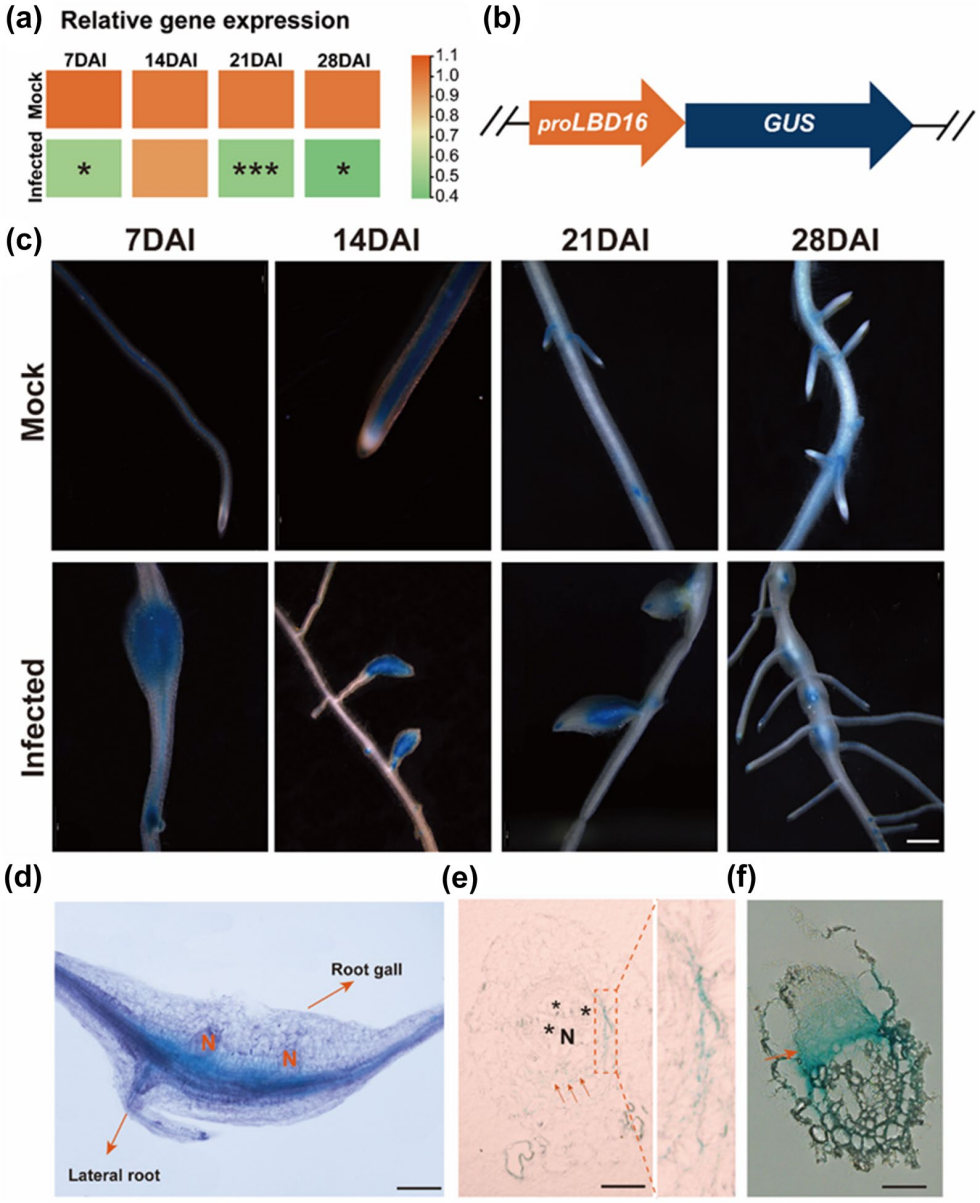
Expression of *pCsLBD16*::*GUS* pattern was activated in cucumber transgenic hairy roots during *M. incognita* parasitism. **a** Relative expression of *CsLBD16* gene in mock-infection and nematode-infection roots during nematode parasitism. Student’s *t*-test was used for statistical analysis. n = 4. *P < 0.05, ***P < 0.001. **b** Contruction of pCAMBIA 1391 vector carrying the promoter region of *CsLBD16.* **c** Transgenic hairy roots harboring *pCsLBD16::GUS* expression pattern with mock-infection and *M. incognita*-infection at 7, 14, 21 and 28 DAI, Bar:500 μm. **d** Expression of the *pCsLBD16::GUS* reporter gene was strengthened in root galls around the head of female nematode. N, nematodes; Bar: 500 μm. **e** Fresh frozen sections of galls from *pCsLBD16*::*GUS* at 28 DAI showed *GUS* staining inside the pericycle cells around giant cells. Asterisks, GCs; N, nematodes; Bar: 200 μm. **f** Paraffin sections of cucumber transgenic hairy roots showed the concentrated *GUS* staining at the base of the lateral root primordium. Arrows indicated *GUS* staining in the lateral root primordium. Bar: 200 μm

The *LATERAL ORGAN BOUNDARIES-DOMAIN* (*LBD16*) is a potential host susceptibility gene for *Meloidogyne javanica* in Arabidopsis [38]. Expression of *CsLBD16* gene significantly reduced in cucumber roots at 7, 21 and 28 DAI after *M. incognita* infection (Fig. 5a). To determine the response of *CsLBD16* to nematode parasitism, a 1391-*pCsLBD16::GUS* reporter vector containing 2000 bp of the *CsLBD16* promoter region was transformed into *R. rhizogenes strain* K599 to induce transgenic hairy roots using the PCI method (Fig. 5b). In the transgenic hairy roots by mock-infection, *pCsLBD16::GUS* was strongly expressed in vascular cylinder and root cap of young roots at 7 and 14 DAI (Fig. 5c). With the growth of mock-infected transgenic hairy roots, only the base of lateral roots showed obvious *pCsLBD16::GUS* expression at 21 and 28 DAI (Fig. 5c). By contrast, the *M. incognita*-infected transgenic hairy roots showed a strong expression of *pCsLBD16::GUS* in root galls at 7 and 14 DAI (Fig. 5c). However, the intensity of *GUS* staining decreased at 21and 28 DAI in the *M. incognita* infected transgenic hairy roots (Fig. 5c). It was noted that *pCsLBD16::GUS* was expressed mainly in the center of galls at 28 DAI (Fig. 5c).

The root galls was compressed with chloral hydrate solution showed that *CsLBD16* expression was induced in the gall surrounding the nematode head through *pCsLBD16::GUS* staining (Fig. 5d). In addition to the gall, the lateral root developing on the gall also showed strong *GUS* staining (Fig. 5d). It is particularly noted that the *GUS* signal was detected around the giant cell (Fig. 5e) and the lateral root primordium (Fig. 5f), indicating that there was a connection between root gall development and lateral root emergence in cucumber roots.

## Discussion

Genetic transformation is generally labor-intensive and time-consuming for many cucurbitaceous plants [39, 40], which usually take 1–2 year to obtain stable transgenic plants. In this study, we developed a peat-based cotyledon-node injection (PCI) method that can rapidly and efficiently induce stable transgenic hairy roots in cucumber, an economically important cultivated crop belonging to Cucurbitaceae family [29].

Particularly, the PCI method allows generation of stable transgenic hairy roots in living plants within only 1–2 months (Figs. 1c and 2). Although previous studies have demonstrated that stable transgenic hairy roots can be induced in coffee by using the solid-medium-based hypocotyl-cutting infection (SHI) method [41] and in tobacco and tomato by using the rockwool-based hypocotyl-cutting infection (RHI) method [42], our study demonstrated that the PCI method overall outperformed the SHI and RHI methods, because the SHI method stimulated less stable transgenic roots as compared to the PCI method (Fig. 2d, e), while the RHI method-induced hairy roots were tightly bound on the rockwool making it hard to measure the transgenic hairy roots (Fig. 2a, b). More importantly, the PCI method allows injection of bacterial mass into the cotyledon-node, which can directly induce hairy roots that are suitable for fluorescent screening and nematode infection (Fig. 1c). Transgenic hairy roots enable the accomplishment of nematode parasitic cycle [26, 41, 43] and the functional analysis of the promoter for the target gene [44, 45], inferring the capacity of the PCI method to explore candidate genes associated with nematode parasitism.

The PCI method can be effectively used for CRISPR/Cas9-mediated genome editing in hairy roots, as demonstrated by the *CsMS* gene associated with nematode parasitism as a case study (Figs. 3 and 4). To the best of our knowledge, this is the first report on the editing of genes encoding malate synthase (MS) which is closely associated with nematode parasitism. The MS is a key enzyme responsible for malic acid synthesis in the glyoxylate cycle [46] and may be involved in plant response to abiotic stresses (e.g. salinity and high temperature [47]). However, little information is available regarding the role of MS in plant response to biotic stresses (e.g. RKN infection). Nematode infection stimulates root metabolism due to the requirement of nutrients by nematode for the establishment of nematode feeding sites [48]. In plant cells, malic acid, whose synthesis is catalyzed by MS [46], is a substance that links various metabolisms of different organelles and participates in metabolic reactions [49]. Reduction of *CsMS* expression after nematode infection indicated the disruption of glyoxylate cycle at early stage of nematode parasitism (Fig. 3a). The *CsMS* gene expression is repressed in roots where the sucrose is actively imported [50] which may consistent with the condition of the sucrose flow to the giant cells from the phloem [51]. The CRISPR/Cas9-mediated knockout *CsMS* gene in hairy roots resulted in a significant reduction in the number of both galls and egg masses (Fig. 4h, i). Collectively, a significant developmental delay was observed in the CRISPR/Cas9-guided *CsMS* knock-out hairy roots due to the disruption of feeding site establishment (Fig. 4a–f). Therefore, the *CsMS* gene in glyoxylic acid cycle responded to nematode infection and the rearrangement of the metabolic activities of root tissues. There is also evidence that plant-growth-promoting rhizobacteria (e.g. *Bacillus subtilis*) can induce biosynthesis of malic acid in plant roots [52], suggesting that the *MS* gene may play an important role in the interaction between roots and microbes. Taken together, the PCI method-induced transgenic hairy roots system offers the opportunity to explore the function of candidate genes associated with nematode parasitism.

In addition to candidate genes, root system development is another aspect that is frequently concerned by studies related to root-nematode interaction [38, 53]. Due to the similar development patterns shared by lateral root development and RKN gall formation [54], we selected the Arabidopsis *AtLBD16* homologous gene *CsLBD16*, which may play an important role in the development of the cucumber root system, to measure host response to nematode parasitism. Clearly, *M. incognita* infection induced the expression of the *GUS* gene in cucumber roots, especially in the gall (Fig. 5c). The root primordium and the pericycle cells showed strong *GUS* signals in the paraffin sections, indicating that *CsLBD16* gene was involved in the initiation of cucumber root primordium (Fig. 5d, f). A previous study showed the transcription factor *AtLBD16* played an important role in both lateral root formation and *M. javanica*-induced gall development in Arabidopsis [38]. The giant cells in the gall result from nuclear divisions of vascular cells without cytokinesis [8]. The activation of *AtLBD16* expression leads to organ initiation via promotion of cell division and establishment of root-primordium identity in pericycle cells [55]. It has been demonstrated that *AtLBD16* participates in the auxin signaling cascade leading to the division of specific XPP cells to form the new organ [56], and induces the formation of nematode permanent feeding site in the pericycle/endodermis [57]. Similarly, in this study, the GUS signal detected around both the giant cell (Fig. 5e) and the lateral root primordium (Fig. 5f) confirmed the connection between lateral root emergence and root gall development in cucumber roots after *M. incognita* infection.

## Conclusion

In conclusion, the transgenic hairy roots induced by the PCI method can replace the original roots to sustain cucumber growth and enable nematode parasitism. This method forms a rapid and efficient tool for easily exploring interactions between cucumber root and RKNs, making it possible to study of root development and the establishment of feeding sites during nematode parasitism. Moreover, the PCI method allows fast, easy and efficient in vivo studies of potential genes related to root-knot nematode parasitism and host response.

## Supplementary Information

**Additional file 1: Figure S1. **Sequence of *CsMS *gene. **Figure S2. **Root galls of transgenic hairy roots under a fluorescence microscope.

## Data Availability

Not applicable.

## References

[1] Phani V, Khan MR, Dutta TK (2021). Plant-parasitic nematodes as a potential threat to protected agriculture: current status and management options. Crop Prot.

[2] Jones JT, Haegeman A, Danchin EGJ, Gaur HS, Helder J, Jones MGK, Kikuchi T, Manzanilla-López R, Palomares-Rius JE, Wesemael WML, Perry RN (2013). Top 10 plant-parasitic nematodes in molecular plant pathology. Mol Plant Pathol.

[3] Peiris PUS (2021). Use of botanicals in root-knot nematode control: a meta-analysis. J Plant Dis Prot.

[4] Holbein J, Franke RB, Marhavý P, Fujita S, Górecka M, Sobczak M, Geldner N, Schreiber L, Grundler FMW, Siddique S (2019). Root endodermal barrier system contributes to defence against plant-parasitic cyst and root-knot nematodes. Plant J.

[5] Saucet SB, Van Ghelder C, Abad P, Duval H, Esmenjaud D (2016). Resistance to root-knot nematodes *Meloidogyne* spp. in woody plants. New Phytol.

[6] Mertens D, Boege K, Kessler A, Koricheva J, Thaler JS, Whiteman NK, Poelman EH (2021). Predictability of biotic stress structures plant defence evolution. Trends Ecol Evol.

[7] Warmerdam S, Sterken MG, Van Schaik C, Oortwijn ME, Sukarta OC, Lozano-Torres JL, Dicke L, Helder J, Kammenga JE, Goverse A, Bakker J, Smant G (2018). Genome-wide association mapping of the architecture of susceptibility to the root-knot nematode *Meloidogyne incognita* in Arabidopsis thaliana. New Phytol.

[8] Mejias J, Bazin J, Truong NM, Chen Y, Marteu N, Bouteiller N, Sawa S, Crespi MD, HervéVaucheret H, Abad P, Favery B, Quentin M (2021). The root-knot nematode effector MiEFF18 interacts with the plant core spliceosomal protein SmD1 required for giant cell formation. New Phytol.

[9] Dong J, Zielinski RE, Hudson ME (2020). t-SNAREs bind the Rhg1 α-SNAP and mediate soybean cyst nematode resistance. Plant J.

[10] Hada A, Kumari C, Phani V, Singh D, Chinnusamy V, Rao U (2020). Host-induced silencing of FMRFamide-like peptide genes, flp-1 and flp-12, in rice impairs reproductive fitness of the root-knot nematode *Meloidogyne graminicola*. Front Plant Sci.

[11] Dong J, Hudson ME (2022). WI12 Rhg1 interacts with DELLAs and mediates soybean cyst nematode resistance through hormone pathways. Plant Biotechnol J.

[12] Zupan J, Muth TR, Draper O, Zambryski P (2000). The transfer of DNA from *Agrobacterium tumefaciens* into plants: a feast of fundamental insights. Plant J.

[13] Jupe F, Rivkin AC, Michael TP, Zander M, Motley ST, Sandoval JP, Slotkin RK, Chen H, Castanon R, Nery JR, Ecker JR (2019). The complex architecture and epigenomic impact of plant T-DNA insertions. PLoS Genetics.

[14] Milligan SB, Bodeau J, Yaghoobi J, Kaloshian I, Zabel P, Williamson VM (1998). The root knot nematode resistance gene Mi from tomato is a member of the leucine zipper, nucleotide binding, leucine-rich repeat family of plant genes. Plant Cell.

[15] Huang G, Allen R, Davis EL, Baum TJ, Hussey RS (2006). Engineering broad root-knot resistance in transgenic plants by RNAi silencing of a conserved and essential root-knot nematode parasitism gene. Proc Natl Acad Sci.

[16] Ibrahim HM, Alkharouf NW, Meyer SL, Aly MA, Abd El Kader Y, Hussein EH, Matthews BF (2011). Post-transcriptional gene silencing of root-knot nematode in transformed soybean roots. Exp Parasitol.

[17] Grossi-de-Sa M, Petitot AS, Xavier DA, Sá MEL, Mezzalira I, Beneventi MA, Martins NF, Baimey HK, Albuquerque EVS, Grossi-de-Sa MF, Fernandez D (2019). Rice susceptibility to root-knot nematodes is enhanced by the *Meloidogyne incognita* MSP18 effector gene. Planta.

[18] Anjanappa RB, Gruissem W (2021). Current progress and challenges in crop genetic transformation. J Plant Physiol.

[19] Ron M, Kajala K, Pauluzzi G, Wang D, Reynoso MA, Zumstein K, Garcha J, Winte S, Masson H, Inagaki S, Federici F, Sinha N, Deal RB, Bailey-Serres J, Brady SM (2014). Hairy root transformation using *Agrobacterium rhizogenes* as a tool for exploring cell type-specific gene expression and function using tomato as a model. Plant Physiol.

[20] Irigoyen S, Ramasamy M, Pant S, Niraula P, Bedre R, Gurung M, Rossi D, Laughlin C, Gorman Z, Achor D, Levy A, Kolomiets MV, Sétamou M, Badillo-Vargas IE, Avila CA, Irey MS, Mandadi KK (2020). Plant hairy roots enable high throughput identification of antimicrobials against *Candidatus Liberibacter* spp. Nat Commun.

[21] Cheng Y, Wang X, Cao L, Ji J, Liu T, Duan K (2021). Highly efficient *Agrobacterium rhizogenes*-mediated hairy root transformation for gene functional and gene editing analysis in soybean. Plant Methods.

[22] Triozzi P, Schmidt HW, Dervinis C, Kirst M, Conde D (2021). Simple, efficient and open-source CRISPR/Cas9 strategy for multi-site genome editing in *Populus tremula* × alba. Tree Physiol.

[23] Giri A, Narasu ML (2000). Transgenic hairy roots: recent trends and applications. Biotechnol Adv.

[24] Xue R, Wu X, Wang Y, Zhuang Y, Chen J, Wu J, Ge W, Wang L, Wang S, Blair MW (2017). Hairy root transgene expression analysis of a secretory peroxidase (*PvPOX1*) from common bean infected by Fusarium wilt. Plant Sci.

[25] Li X, Qin R, Du Q, Cai L, Hu D, Du H, Yang H, Wang J, Huang F, Wang H, Yu D (2020). Knockdown of *GmVQ58* encoding a VQ motif-containing protein enhances soybean resistance to the common cutworm (*Spodoptera litura Fabricius*). J Exp Bot.

[26] Yang Y, Jittayasothorn Y, Chronis D, Wang X, Cousins P, Zhong GY (2013). Molecular characteristics and efficacy of 16D10 siRNAs in inhibiting root-knot nematode infection in transgenic grape hairy roots. PloS ONE.

[27] Rechenmacher C, Wiebke-Strohm B, de Oliveira-Busatto LA, Weber RLM, Corso MCM, Lopes-Caitar VS, Silva SMH, Dias WP, Marcelino-Guimarães FC, Carlini CR, Bodanese-Zanettini MH (2019). Endogenous soybean peptide overexpression: an alternative to protect plants against root-knot nematodes. Biotechnol Res Innov.

[28] Pereira BM, Guimaraes LA, Souza NO, Saraiva MA, Guimaraes PM, Brasileiro AC (2019). Overexpression of wild Arachis lipocalin enhances root-knot nematode resistance in peanut hairy roots. Plant Mol Biol Report.

[29] Huang S, Li R, Zhang Z, Li LI, Gu X, Fan W (2009). The genome of the cucumber, *Cucumis sativus* L. Nat Genet.

[30] Walters SA, Wehner TC, Barkel KR (1993). Root-knot nematode resistance in cucumber and horned cucumber. HortScience.

[31] Mukhtar T, Kayani MZ, Hussain MA (2013). Response of selected cucumber cultivars to *Meloidogyne incognita*. Crop Prot.

[32] Hu B, Li D, Liu X, Qi J, Gao D, Zhao S, Huang S, Sun J, Yang L (2017). Engineering non-transgenic gynoecious cucumber using an improved transformation protocol and optimized CRISPR/Cas9 system. Mol Plant.

[33] Liu M, Liang Z, Aranda MA, Hong N, Liu L, Kang B, Gu Q (2020). A cucumber green mottle mosaic virus vector for virus-induced gene silencing in cucurbit plants. Plant Methods.

[34] Wang ZP, Xing HL, Dong L, Zhang HY, Han CY, Wang XC, Chen QJ (2015). Egg cell-specific promoter-controlled CRISPR/Cas9 efficiently generates homozygous mutants for multiple target genes in Arabidopsis in a single generation. Genome Biol.

[35] Xie X, Ma X, Zhu Q, Zeng D, Li G, Liu YG (2017). CRISPR-GE: a convenient software toolkit for CRISPR-based genome editing. Mol Plant.

[36] Meng D, Yang Q, Dong B, Song Z, Niu L, Wang L, Cao H, L, H., Fu, Y. (2019). Development of an efficient root transgenic system for pigeon pea and its application to other important economically plants. Plant Biotechnol J.

[37] Bybd DW, Kirkpatrick T, Barker KR (1983). An improved technique for clearing and staining plant tissues for detection of nematodes. J Nematol.

[38] Cabrera J, Díaz-Manzano FE, Sanchez M (2014). A role for LATERAL ORGAN BOUNDARIES-DOMAIN 16 during the interaction Arabidopsis-Meloidogyne spp. provides a molecular link between lateral root and root-knot nematode feeding site development. New Phytol.

[39] Fang L, Wei XY, Liu LZ, Zhou LX, Tian YP, Geng C, Li XD (2021). A tobacco ringspot virus-based vector system for gene and microRNA function studies in cucurbits. Plant Physiol.

[40] Nishibayashi S, Hayakawa T, Nakajima T, Suzuki M, Kaneko H (1996). CMV protecton in transgenic cucumber plants with an introduced CMV-O cp gene. Theor Appl Genet.

[41] Alpizar E, Dechamp E, Espeout S (2006). Efficient production of *Agrobacterium rhizogenes*-transformed roots and composite plants for studying gene expression in coffee roots. Plant Cell Rep.

[42] Collier R, Fuchs B, Walter N, Kevin Lutke W, Taylor CG (2005). Ex vitro composite plants: an inexpensive, rapid method for root biology. Plant J.

[43] Cho HJ, Farrand SK, Noel GR, Widholm JM (2000). High-efficiency induction of soybean hairy roots and propagation of the soybean cyst nematode. Planta.

[44] Fan YL, Zhang XH, Zhong LJ, Wang XY, Jin LS, Lyu SH (2020). One-step generation of composite soybean plants with transgenic roots by *Agrobacterium rhizogenes*-mediated transformation. BMC Plant Biol.

[45] Van Nguyen D, Hoang TTH, Le NT, Tran HT, Nguyen CX, Moon YH, Do PT (2021). An efficient hairy root system for validation of plant transformation vector and CRISPR/Cas construct activities in cucumber (*Cucumis sativus L*.). Front Plant Sci.

[46] Pua EC, Chandramouli S, Han P, Liu P (2003). Malate synthase gene expression during fruit ripening of Cavendish banana (*Musa acuminata* cv Williams). J Exp Bot.

[47] Brito VC, de Almeida CP, Barbosa RR (2020). Overexpression of *Ricinus communis* L. malate synthase enhances seed tolerance to abiotic stress during germination. Ind Crops Prod.

[48] Hofmann J, El Ashry AEN, Anwar S, Erban A, Kopka J, Grundler F (2010). Metabolic profiling reveals local and systemic responses of host plants to nematode parasitism. Plant J.

[49] Scheibe R (2004). Malate valves to balance cellular energy supply. Physiol Plant.

[50] Graham IA, Leaver CJ, Smith SM (1992). Induction of malate synthase gene expression in senescent and detached organs of cucumber. Plant Cell.

[51] Xu LH, Xiao LY, Xiao YN, Peng DL, Xiao XQ, Huang WK, Gheysen G, Wang GF (2021). Plasmodesmata play pivotal role in sucrose supply to Meloidogyne graminicola-caused giant cells in rice. Mol Plant Pathol.

[52] Rekha K, Baskar B, Srinath S, Usha B (2018). Plant-growth-promoting rhizobacteria Bacillus subtilis RR4 isolated from rice rhizosphere induces malic acid biosynthesis in rice roots. Can J Microbiol.

[53] Bird DM, Opperman CH, Williamson VM (2009). Plant infection by root-knot nematode. In cell biology of plant nematode parasitism.

[54] Mathesius U, Abe Jun (2003). Conservation and divergence of signalling pathways between roots and soil microbes—the Rhizobium-legume symbiosis compared to the development of lateral roots, mycorrhizal interactions and nematode-induced galls. Roots The dynamic interface between plants and the earth.

[55] Liu W, Yu J, Ge Y, Qin P, Xu L (2018). Pivotal role of LBD16 in root and root-like organ initiation. Cell Mol Life Sci.

[56] Goh T, Joi S, Mimura T (2012). The establishment of asymmetry in Arabidopsis lateral root founder cells is regulated by LBD16/ASL18 and related LBD/ASL proteins. Development.

[57] Bartlem DG, Jones MGK, Hammes UZ (2014). Vascularization and nutrient delivery at root-knot nematode feeding sites in host roots. J Exp Bot.

