# Supplementary material for: Peat-based hairy root transformation using Rhizobium rhizogenes as a rapid and efficient tool for easily exploring potential genes related to root-knot nematode parasitism and host response
Source: Plant Methods. 2023 Mar 4;19:22. doi: 10.1186/s13007-023-01003-3 (PMC9985853; doi:10.1186/s13007-023-01003-3)
Supplement: Supplementary file 1 — Additional file 1: Figure S1. Sequence of CsMS gene. Figure S2. Root galls of transgenic hairy roots under a fluorescence microscope. [file 13007_2023_1003_MOESM1_ESM.pdf]

Figure.S1 Sequence of *CsMS* gene

ATGGGATCGCTTGGGATGTATTCCGAATCAGGATTAACGAAGAAGGGTAGCAGCAGAGGCTACGACGT
1 Target 1
TCCAGAGGGAGTGGACATTCGAGGACGCTATGATGAAGAATTTGCCAAAATTCTCAACAAGGAAGCC
TTGTTGTTTGTCGCTGATTTACAGAGAACTTTTAGAAACCACATCAAGTATTCGATGGAATGCCGCAGA
GAAGCCAAGAGGCGGTATAATGAAGGGGGGTTACCGGGGTTTGATCCGGCGACTAAGTATATAAGAGA
TTCTGAGTGGACATGTGCCCCTGTCCCCCCAGCAGTTGCTGATCGGAGAGTGGAGATCACCGGACCTG
TGGAGCGGAAGATGATCATCAACGCACTCAATTCTGGAGCTAAAGTTTTCATGGTCTGTTCTCTCTTCC
TTCTCTCTCCTAAAGACTTTGTTTTTATTTAAGATCTTGAAATGTCTGGAGTATGTTGTCTGTCCCACTG
CCCCTCAGAACAATCAAAAACTTGTTCTTTTTAATAACAAACCGTTTGTAGACAGTAGACAGGCATGA
GCTCGAGCTGGTGTAGTGGGATGAATAACAATTTTAGGGCAGGTATTATTTCAGTTATATATATATATATA
TATATTATATTAAAAGCATGAAGTCCGCGCCATGGGCCTATACTAAATCAGTCCGTGTAGATAGCACTTA
AAGCACTGTTATGGAACTAAGTTTCAATGTCTATTCTATGCAATTTGCATTTATTTGCTTTTCGTAACGTG
GATTTCATGACATAAAAGAATGAAGTTTGATGAAAAAACCAATCAAAATGAAAACAGGCGGACTTTGA
AGATGCACTGTCACCAAATTGGGAGAATCTGATGAGGGGGCAAATTAATCTTAAGGATGCAGTGGATG
GGACTATAAGTTTCCATGATAGAGTTAGAAACAGGGTTTATAAGCTGAATGATCAGACAGCCAAGCTCT
TTGTCCGCCCTCGAGGTTGGCATTTGCCAGAGGCTCATATCTTCATTGACGGCGAGCCTGCAACCGGCT
GTCTTGTGGATTTTGGGCTCTATTTTTTCCACAACCATGCTAATTTTCGACGCTCTCAAGGTCAAGGTTA
Target 2
TGGCCCTTTCTTTTACCTTCCCAAAATGGAGCACTCCAGGTAAATCTTCATCCAAATATTATTATGTAAC
CAATTGTGCAAGTTTTGAGAAATTGAGCTCTGTTTGCAATGAGAGTCGCTTGTAGATGATGCATAATTG
TCCCTGATAAAATAGGCTACAAGATAACATTATGTAAAGAATGAACACCCCTGGAAAATAGACGTTAGT
TTGAGGTTAAAAACTACTCTGTTTTGAGAATTAGTCTCGTGTCTAGAAAGTATAGATCCTATAAACTTAA
TCTAATCATCTAAGACTTGCAAGAAAAGTCTCTTTTGAAAGCCAGATTCAAGAGCCTTTTTACCTGGGT
CATAATAAATTCAGATCACCACATTCAAACAATCTGATTGGAAGAGTTATTAGACAATTAGTATATTTTA
GTTAATATTTGTTTAGTTGTTTATTTAGATTAGTATTATTCTAGAATTAATGAGTTAGTTATCTTATTTTCTA
GCCATTTTAGGGAGTTTTTAAAATATGATGTTGTTCTTGGAGAGTTCTCTCAACTTAGGGATGGACTTTT
CTTGTTTGACCATGAATTTGGCCTACATCACAATCTTACGAACGGTCTGGTCTAATATGTGATGTGTAGG
GAAGCTAAAATATGGAACAGTGTATTTGAGAGGGCAGAGAAGATGGCAGGGATAGAGAGGGGCAGCA
TCAGGGCCACCGTACTAATTGAAACACTTCCAGCAGTGTTTCAAATGAATGAAATACTTTACGAGCTG
AGGGATCACTCTGTGGGATTGAACTGTGGTAGATGGGATTACATATTCAGCTATGTCAAGACCTTCCAG
GCTCACCCAGATCGTCTATTACCCGATCGGGTCCTAGTCGGTATGACCCAACATTTCATGAGGAGCTAT
TCTGATCTTCTCATCAGGACTTGTCACAGGCGTGGTGTGCATGCCATGGGAGGCATGGTAGGTATAATA
CAAACATTGCATTCAATAATATTTCACATCCAGAGTTTTGACAGCTAGATTCTTTTTGCAGGCTGCTCAA
ATTCCGATTAGAGACGATCCGAAGGCAAATGAGGTAGCACTTGAGCTAGTGAGGAAGGACAAACTGA
GGGAGGTAAAGGCAGGGCATGATGGAACATGGGCAGCACATCCAGGACTAATCCCGGCTTGTATGGA
AGTCTTCACCAACAACATGGGGAATGCCCCCAACCAAATTCGATCCATGAGACGTGACGATGCTGCAA
ACTTGACCGAAGAAGATCTCTTACAACGACCAAGAGGTGTACGTACAATGGAAGGGCTTCGCTTGAA
CACCCGAGTTGGGATTCAGTACCTAGCCGCATGGCTAACCGGGGCTGGCTCGGTGCCTCTATACAACC
TTATGGAAGATGCAGCAACAGCAGAAATTAGCAGGGTTCAAAACTGGCAATGGCTGAAGTATGGAGT
GGAATTGGATGGAGATGGGCTTGGTGTGAGAGTGAACAAGGAACTGTTTGGGAGAGTGGTGGAAGA
AGAAATGGAAAGGATTGAAAGAGAAGTTGGGAAAGAGAGATTCAAAAAAGGAATGTACAAAGAAGC
TTGCAAGATGTTCACAAGGCAATGCACAGCTCCAAATTTGGATGACTTTCTGACCTTAGACGCTTACA
ACTATATTGTTATACATCATCCAAGGGAATTGTCCAAGCTT**TGA**
2793

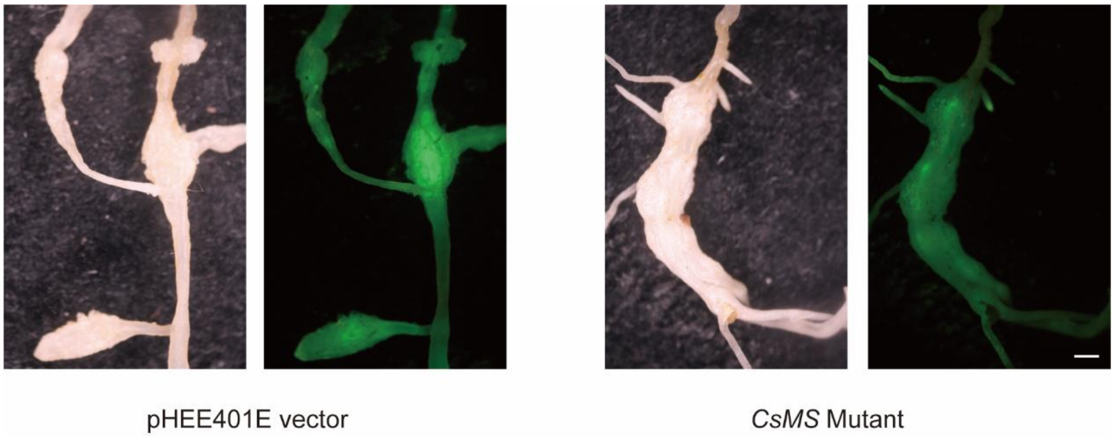

Figure S2 Root galls of transgenic hairy roots under a fluorescence microscope
